# Effect of a UV-C Automatic Last-Generation Mobile Robotic System on Multi-Drug Resistant Pathogens

**DOI:** 10.3390/ijerph182413019

**Published:** 2021-12-10

**Authors:** Carla Russo, Desirée Bartolini, Cristina Corbucci, Anna Maria Stabile, Mario Rende, Antimo Gioiello, Gabriele Cruciani, Antonella Mencacci, Francesco Galli, Donatella Pietrella

**Affiliations:** 1 Microbiology and Clinical Microbiology Section, Department of Medicine and Surgery, University of Perugia, 06129 Perugia, Italy; carla.russo@studenti.unipg.it (C.R.); antonella.mencacci@unipg.it (A.M.); 2 Unit of Human, Clinical and Forensic Anatomy, Department of Medicine and Surgery, University of Perugia, 06129 Perugia, Italy; desirex85@hotmail.it (D.B.); anna.stabile@unipg.it (A.M.S.); mario.rende@unipg.it (M.R.); 3 Microbiology Unit, Santa Maria della Misericordia Hospital, 06129 Perugia, Italy; cristina.corbucci@ospedale.perugia.it; 4 Department of Pharmaceutical Sciences, University of Perugia, 06122 Perugia, Italy; antimo.gioiello@unipg.it; 5 Department of Chemistry, Biology and Biotechnology, University of Perugia, 06123 Perugia, Italy; gabriele.cruciani@unipg.it

**Keywords:** UV-C light device, MDR microorganisms, photoreactivation, high-touch surface, disinfection

## Abstract

Background: Healthcare-associated infections caused by multi-drug resistant (MDR) pathogens are associated with increased mortality and morbidity among hospitalized patients. Inanimate surfaces, and in particular high-touch surfaces, have often been described as the source for outbreaks of nosocomial infections. The present work aimed to evaluate the efficacy of a last-generation mobile (robotic) irradiation UV-C light device R2S on MDR microorganisms in inanimate surfaces and its translation to hospital disinfection. Methods: The efficacy of R2S system was evaluated in environmental high-touch surfaces of two separate outpatient rooms of Perugia Hospital in Italy. The static UV-C irradiation effect was investigated on both the bacterial growth of *S. aureus*, MRSA, *P. aeruginosa*, and *K. pneumoniae* KPC and photoreactivation. The antimicrobial activity was also tested on different surfaces, including glass, steel, and plastic. Results: In the environmental tests, the R2S system decreased the number of bacteria, molds, and yeasts of each high-touch spot surface (HTSs) compared with manual sanitization. UV-C light irradiation significantly inhibits in vitro bacterial growth, also preventing photoreactivation. UV-C light bactericidal activity on MDR microorganisms is affected by the type of materials of inanimate surfaces. Conclusions: The last-generation mobile R2S system is a more reliable sanitizing procedure compared with its manual counterpart.

## 1. Introduction

Hospital-associated infections caused by multidrug-resistant microorganisms are one of the biggest threats to public health in the EU/EEA region and globally. Among MDR pathogens, methicillin-resistant *Staphylococcus aureus* (MRSA), carbapenemases producing *Klebsiella pneumoniae* (KPC), and *Pseudomonas aeruginosa* are often isolated [1]. Ears-Net (European Antimicrobial Resistance Surveillance Network) reported more than 670,000 infections/year in the EU/EEA due to bacteria’s resistance to antibiotics, and that approximately 33,000 people die as a direct consequence of these types of infection. The related cost to the healthcare systems of EU/EEA countries is around EUR 1.1 billion [2]. The major sources of healthcare-associated infection pathogens are endogenous microorganisms and those related to contaminated environmental surfaces [3]. High-touch surfaces (HTSs), such as telephones, computers, and keyboards have been considered potential vectors for transmitting nosocomial pathogens [4]. Most Gram-positive bacteria, such as *Enterococcus* spp. (including VRE), MRSA or *Streptococcus pyogenes*, and many Gram-negative species, such as *Acinetobacter* spp., *Escherichia coli*, *Klebsiella* spp., *P. aeruginosa*, *Serratia marcescens*, or *Shigella* spp., can survive for months on dry surfaces; *Candida albicans*, the most important nosocomial fungal pathogen, can survive up to 4 months [1].

Standard approaches of surface cleaning are often suboptimal in eliminating environmental pathogens; the inadequate decontamination of hospital surfaces has driven the development of room cleaning devices. Ultraviolet-C light (UV-C) decontaminating devices are increasingly added to standard cleaning and disinfection in healthcare facilities. UV-C devices (254 nm) have been used to decontaminate patients’ rooms and hospital facilities. New systems have been designed to operate as robotic devices with automated and trackable procedures [5,6]. Recent applications of this type of device have also been extended to COVID-19 patients’ room decontamination [7]. Mobile devices are expected to overcome the limits of efficacy of fixed UV-C systems that include a series of physical barriers to optimal irradiation of the different surfaces in the environment (for example, the propagation of light intensity decreases exponentially with increasing distance from the lamp, light angle projection and reflection of surfaces may interfere) [8]. Many robots and fixed systems show UV-C lamps arranged in a tower. This structure is a physical limit for the UV-C irradiation that needs much more energy to emit the intensity needed to produce the germicidal UV-C dose. The “super-power” of the lamps reduces the autonomy of the system, as well as causes premature aging of the plastic surfaces present in the rooms to be disinfected.

The efficacy of bacterial inactivation by UV-C radiation depends on specific mechanisms of molecular damage, which are expected to be irreversible. Such inactivation is mainly due to DNA damage and the inhibition of DNA replication. UV light induces pyrimidine dimers formation, pyrimidine 6-4 pyrimidone photoproducts (6-4 PPs), and their Dewar isomers. However, some bacteria can repair the damaged DNA after UV irradiation by photoreactivation [9]. Photoreactivation is a natural process in which bacterial cells can partially recover from the UV light damage by visible and UV wavelengths light. The process is catalyzed by enzymes present in many cells and it occurs in some bacteria and spores; conversely, viruses have a very limited self-repair ability. In the photoreactivation process, microorganisms utilize light in the wavelength range of 330–480 nm to activate a photolyase enzyme, which binds specifically to 6-4 PPs and directly monomerizes the cyclobutane ring [10].

Recently, a new mobile UV-C irradiation device (Robotic System 2, R2S) has been developed to further improve the irradiation of “blinded”, or partially hidden, surfaces, i.e., surfaces that cannot be directly reached with the optics of first-generation robotic systems and even more with fixed systems. The R2S is equipped with software to plan the disinfection process of different rooms identified with a special QR. Each R2S’s mission consists of a variable number of steps of the UV-C irradiation. The duration of each stop depends on the UV-C dose to be irradiated to inactivate the most resistant pathogen potentially existing in the environment to be disinfected. Once the disinfection process is finished, the R2S robot can communicate and store all the data of the work performed. Data are stored to be unchangeable using a blockchain, a technologically innovative system of recording information. In the present study, the disinfection procedure of hospital outpatient rooms was carried out using the new robotic UV-C irradiation system R2S that was compared with a standard protocol of manual disinfection performed by wiping and nebulization of chlorine-containing solutions. The in vitro antimicrobial effect of UV-C light on different types of bacteria, including MDR species, and the photoreactivation response were investigated. Finally, the inactivation efficacy on bacteria layered on different types of inanimate materials that compose the most common HTSs of nosocomial environments, such as plastics, glass, and steel, were evaluated.

## 2. Materials and Methods

### 2.1. Setting

The study was carried out at “Santa Maria della Misericordia” Hospital, Perugia, Italy, from August to November 2020. The study did not involve research on human subjects.

### 2.2. Robotic System 2 (R2S™)

**Figure 1:**
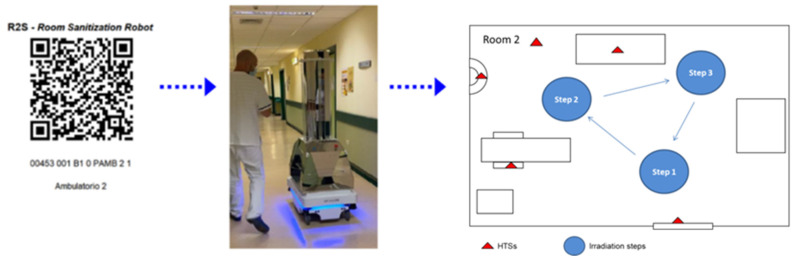
Workflow of the R2S system. R2S robot is equipped with a dedicated app able to read a QR code in which the mission is memorized (all the steps and the time of irradiation of each stop point).

The RS2 (90.0 cm × 59.0 cm × 179 cm—weight: 140–180 kg) is an autonomous mobile robot (AMR) in mirror machined aluminum with a very high capacity of reflecting UV-C radiation (Figure 1). R2S consists of a laser sensors scanner, 3D depth camera, and ultrasonic sensors. The system is equipped with a sensor-based safety shutdown of UV-C lamps on the neural network “Human Body Recognition”. The cooling system is a “closed body” without using air inflow and/or outflow fans. Cooling with forced air would be particularly dangerous given that the forced ventilation system would favor the diffusion of pathogens that would be sucked into the body of the system together with the powder. The lamp-holder tower is equipped with reflecting “octagonal cones” with a high capacity to reflect light in an up–down direction, eight vertical lamps (ozone-free, low-pressure, 254 nm UV-C protected by a quartz case), and four horizontal lamps (ozone-free, low-pressure, 254 nm UV-C). The AppR2S allows the pathway of the robot in the room to be planned and recorded (mission), the map of the room to be identified by a QR code, and the robot to associate the mission to the room (Figure 1).

### 2.3. Microorganisms

*Staphylococcus aureus* American Type Culture Collection (ATCC*^®^*) 25923^TM^ strain and a MRSA 881 clinical isolate were employed to test the efficacy of the UV-C light-emitting device R2S. For experiments with Gram-negative bacteria, PAO-1 (ATCC*^®^* 15692^TM^) and ATCC*^®^* 27853^TM^ *Pseudomonas aeruginosa*, and two *Klebsiella pneumoniae* KPC carbapenemase-producing clinical isolates (KPC 242 and KPC 260) were used. All bacteria strains were streaked for isolation onto agar plates (Muller Hinton Agar, MHA, bioMérieux Italia SpA, Florence, Italy) and a single colony from overnight cultures was inoculated into Muller Hinton Broth (MHB, bioMérieux Italia SpA, Florence, Italy) and cultured at 37 °C. Overnight cultures were suspended in sterile phosphate-buffered saline (PBS). Cell growths were determined by measuring optical densities at 600 nm using a spectrophotometer (Infinite M200pro, TECAN). The bacterial suspensions of *S. aureus*, *P. aeruginosa*, and *K. pneumoniae* were then diluted to the desired final concentrations of 2 × 10^6^/mL, 2 × 10^5^/mL, and 2 × 10^4^/mL.

### 2.4. In Vitro UV-C Irradiation and Photoreactivation

UV-C irradiation treatment was performed with a horizontal lamp, which emits light at the wavelength of 254 nm. The lamp was maintained at a distance of 30 cm the from bacterial cultures and time-course irradiation experiments were performed in duplicate at room temperature, exposing *S. aureus* and *P. aeruginosa* for a span between 10 and 25 s (UV-C exposition time/dose of 4.3 and 12.7 mJ/cm^2^, respectively, determined with a cold lamp, sensor’s temperature T = 27.2 °C), and *K. pneumoniae* for 10 and 40 s (UV-C time/dose of 4.3 and 22.97 mJ/cm^2^, respectively). After UV-C irradiation, a series of plates were kept in the dark for 4 h and then incubated at 37 °C overnight to assess colony forming units (CFU) in the absence of photoreactivation. To evaluate the photoreactivation effect, another series of plates irradiated with UV-C light as described before was exposed to a fluorescent lamp (40 W) for 4 h at room temperature, and then CFU were counted after overnight incubation at 37 °C.

### 2.5. In Vitro Effect of UV-C on Pathogens Layered on Non-Porous Surfaces (Glass, Plastic, Steel)

The bactericidal effect of UV-C light on common materials composing the surfaces to be sanitized in the hospital was evaluated. Ten μL aliquots of bacterial suspensions (2 × 10^8^/mL) of *S. aureus* MRSA, *S. aureus* ATCC*^®^* 25923^TM^, *P. aeruginosa* PAO-1, *P. aeruginosa* ATCC*^®^* 27853^TM^, *K. pneumoniae* KPC242, and KPC260 were spread onto previously sterilized non-porous materials (disks of 55 mm diameter) such as glass, plastic, or steel. Glass and steel surfaces were sterilized in an autoclave, while for plastic experiments sterile petri dishes sterilized by gamma radiation (BD Biosciences) were used. Plates were exposed to UV-C light for 20 s (9.66 mJ/cm^2^) at a distance of 30 cm and, after treatment, the bacteria were recovered using contact plate Petri dishes (55 mm diameter, bioMérieux Italia SpA, Florence, Italy). Preliminary experiments were performed to verify the persistence of bacteria on the surface. After sampling with the RODAC plates swabs of the samples, surfaces were made and spread on MHA plates. The number of colonies obtained was negligible. As a control, in parallel, for each experimental condition, surfaces seeded with the same suspension were covered with a triple layer of aluminum and irradiated. Control circular surfaces were sampled with RODAC plates but CFU were uncountable; swabs of the sample surfaces were made, diluted in MHB, and spread on MHA plates. After sampling, the dishes were incubated at 37 °C overnight and then the number of colonies was evaluated. All experiments were performed in triplicate.

### 2.6. Comparison of Mobile UV-C Irradiation and Conventional Disinfection Procedure

**Figure 2:**
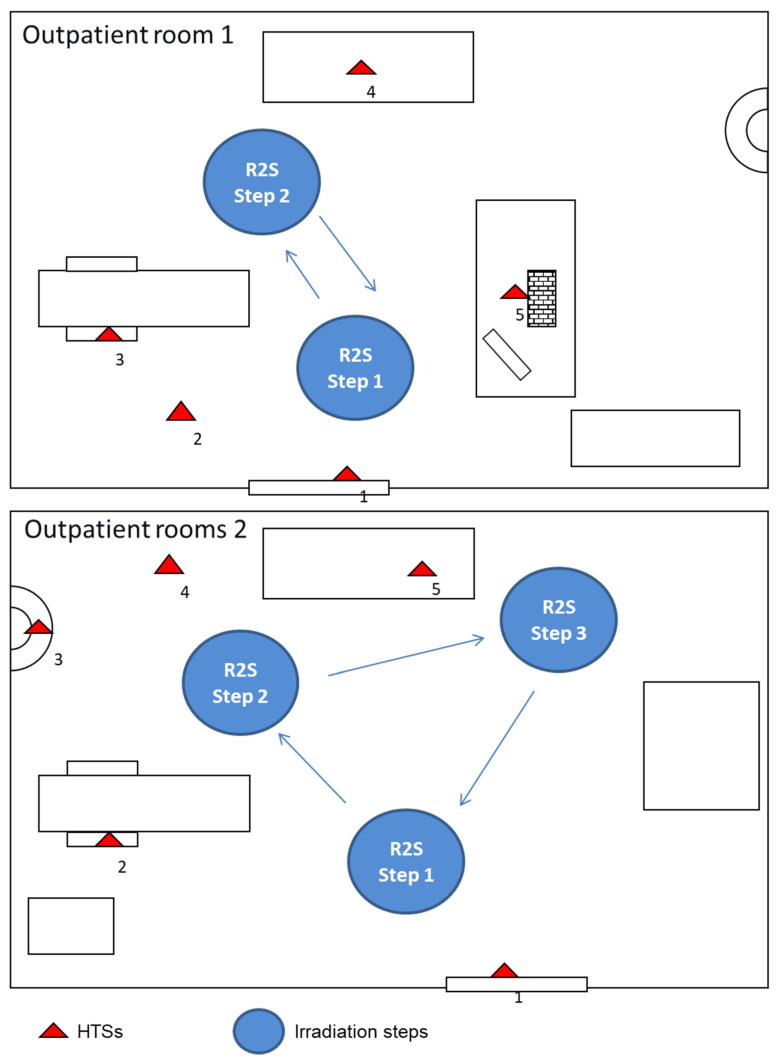
R2S mission performed in two different outpatient rooms (Room 1 and 2) of the “Santa Maria della Misericordia” Hospital in Perugia and the schematic representation of the sampling spots. The test was carried out in the two rooms following the indicated steps. Outpatient rooms 1: the distances measured between the UV-C light device R2S irradiation step 1 and the sampling point of surfaces, such as internal door handle (1), floor (2), the right arm of the patient chair (3), and computer keyboard and mouse (5), which were 28, 45, 62, and 50 cm, respectively. The distance between R2S irradiation step 2 and the sampling spot on the bench (4) was 30 cm. Outpatient rooms 2: the distances measured between the UV-C light device R2S irradiation step 1 and the sampling point of surfaces, such as internal door handle (1) and the right armrest of the patient’s chair (2), which were 25 and 50 cm, respectively. The distances between R2S irradiation step 2 and the sampling points on the sink (3) and floor (4) were 38 and 40 cm, respectively. The distance between R2S irradiation step 3 and the sampling point on the bench (5) was 30 cm.

The bactericidal effect of a new mobile UV-C irradiation device R2S (Bazzica Engineering Srl, Perugia, Italy) [11] was evaluated in comparison with the standard daily sanitization procedure performed at the “Santa Maria della Misericordia” Hospital of Perugia. The R2S device was kindly provided by the manufacturer and a member of this group of researchers (C.R.) was trained and was responsible for remote control of the device and planning of the robotic disinfection sessions. The sanitization routine utilized for comparison with this robotic system consisted of the manual cleaning of furniture and floors, followed by nebulization with chlorine. Manual wiping side-to-side disinfection was performed by using a disposable electrostatic microfiber cloth after the spray application of Gioclorex 0.5% (chlorhexidine digluconate 0.5% and ethanol 70%) for a contact time of 15 s. After manual wiping, the room was nebulized with a chloride solution (1 mg/L) and closed for 15 min to allow the disinfectant to act. The comparative evaluation was performed twice in two consecutive environmental tests carried out in two different outpatient rooms (Figure 2) at the end of the daily activity. The first day, the microbiological control was performed before and after the routine sanification at the end of the work activity; cleaners did not know they were being compared in a research study. The second day the R2S robot was used for disinfection, with the surface control being done before and after the sanification. The R2S was programmed to move around the room during the disinfection mission and the time periods of irradiation were planned. The mission for each room was memorized by the software and used by the robot on different days. The robot was driven into the room by the software AppR2S, the door was closed, and the mission started automatically. The robot is equipped with a security sensor able to recognize a human presence that switches off the lamps immediately in case of entry into the room during the irradiation procedure. In outpatient room 1 (Figure 2), the HTSs examined before and after standard or R2S sanitization were analyzed, including the internal door handle, the right arm of the patient chair, bench, computer keyboard, mouse, and floor. In outpatient room 2 (Figure 2), the internal door handle, right armrest of the patient’s chair (lower and upper surface), bench, sink, and floor were tested. For the R2S sanitation mission, the robot was in outpatient clinic 1 and two irradiation points were chosen: the first irradiation step lasted 360 s and the second 300 s. In outpatient clinic 2, which is larger in size compared with room 1, a third irradiation step of 300 s was added. Microbiologic sampling was performed using contact plate Petri dishes of 55 mm diameter, CT for bacteria, and CTS for molds and yeasts (Biomérieux SA, Marcy-l’Etoile, France) from adjacent non-overlapping surfaces. CT and CTS plates contained four neutralizing agents for inactivating residual chemical disinfectants: the combination of lecithin, polysorbate 80, and L-histidine neutralizes aldehydes and phenolic compounds, the combination of lecithin and polysorbate 80 neutralizes the quaternary ammonium compounds, the polysorbate 80 neutralizes hexachlorophene and mercurial derivates, sodium thiosulfate neutralizes halogen compounds, and lecithin neutralizes chlorhexidine. Contact plates were pressed for 15 s on each surface and then incubated for 48 h at 37 °C (CT) or five days at room temperature (CTS). Preliminary experiments to compare the number of bacteria on non-flat surfaces (keyboard and door handle) recovered by using the RODAC plates or swabs were performed. The number of colonies obtained on RODAC plates was comparable to that obtained by means of swab sampling (Figure S1). Bacterial cultures of the collected samples were prepared in duplicate. To ensure that no accidental contamination was introduced after the sanitization procedure, samplings were performed within 10 min post-sanitization with either chlorine nebulization or the R2S system.

### 2.7. Statistical Analysis

Data are expressed as mean (±SD) of two replicates of three independent experiments. Differences between different groups of data were assessed using either parametric or non-parametric tests that were applied when appropriate after the analysis of data distribution and outliers (95% interval of confidence). Analysis of variance was performed utilizing the ANOVA test.

## 3. Results

### 3.1. R2S UV-C Irradiation Efficacy Compared to Manual Disinfection

The R2S robot has been designed to overcome the limits of fixed UV-C systems which are disadvantaged by the fact that within the environment in which they operate they cannot independently assume positions of UV-C irradiation. In the present study, the robotic UV-C irradiation (R2S system) and manual disinfection procedures have been compared. First of all, the missions of the robot in the rooms (identified by a QR code) were planned and recorded. The mission included the pathway of the R2S robot in the room, the stop points, and the irradiation time periods for each step. R2S was driven into the room, the door was closed, and the mission started. The robot can move by itself in the room, stop in the fixed point, and irradiate 360° of the space around it for the planned time periods (see Supplementary Video S1). The R2S system irradiation and manual disinfection procedures were performed in two outpatient rooms of the local hospital (Figure 2), as described in Materials and Methods.

**Figure 3:**
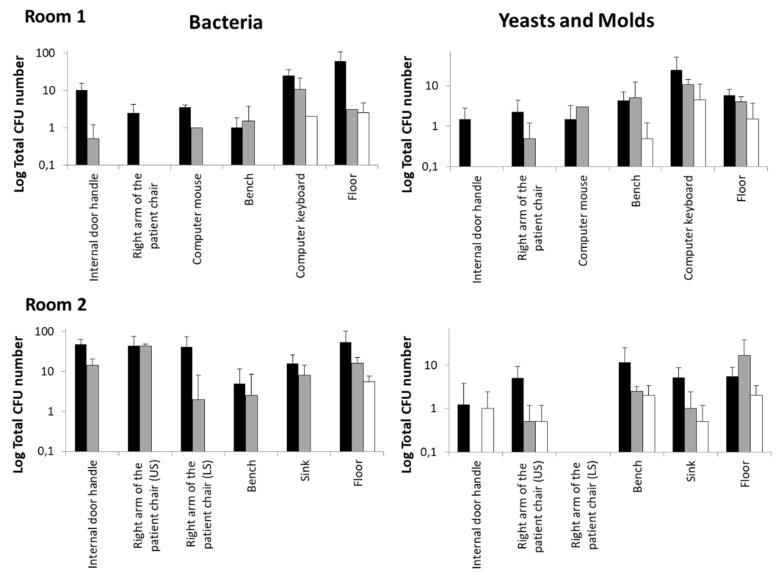
Outpatient room disinfection with the UV-C robotic systems R2S compared to standardized manual protocol based on chlorine solution nebulization. Mean ± SD data were calculated from the two determinations described in Materials and Methods. The CFU number of bacteria, yeast, and molds was assessed as described in the Materials and Methods section. Black bars: CFU before sanification; gray bars: after manual disinfection; white bars: after R2S disinfection.

The microbicidal activity of the R2S system during the sanitization tests of outpatient rooms 1 and 2 was more efficient in comparison with the manual disinfection procedure (Figure 3).

UV-C irradiation reduced the number of bacterial, yeast, and mold CFU in all the sampling spots of the two rooms with an efficacy that was higher, and only in few cases similar, to that observed for the manual protocol. In particular, the contaminating bacteria were completely eliminated by R2S in all surfaces examined except for the computer keyboard of Room 1 and the floor. This result is not surprising if the structure of the keyboard itself and the dust between keys is considered; regardless, the results obtained with UV-C were better than those observed with manual sanitation. Moreover, it is conceivable that the repeated treatment with UV-C of the keyboard could further lower the bacterial load. Regarding the floor, the data obtained with the UV-C and manual sanitation were very similar, probably due to the non-optimal downwards irradiation angle of the R2S.

### 3.2. Bacterial Inactivation and Photoreactivation Test of Static UV-C Irradiation

Given the natural bacterial photoreactivation activity for recovering the induced ultraviolet damage, the photoreactivation effects in MDR microorganisms associated with nosocomial infection with increased mortality and morbidity bacterial strains after exposure to UV-C light have been investigated (Table 1). The irradiation protocol (2.68 mJ/cm^2^ exposure for *S. aureus* and *P. aeruginosa* and 22.97 mJ/cm^2^ for *K. pneumoniae*), chosen according to the literature [12], almost completely inhibited the bacterial growth of all the investigated strains (Table 1). Plates shielded during UV-C treatment were found to maintain several colonies comparable to the number of seeded bacteria (not shown), which confirms the specificity of the irradiation effect on bacterial inactivation. Bacterial concentration-dependence experiments demonstrated that this effect of UV-C light on bacterial growth was rapid and very efficient even when the initial concentrations of the different strains were the highest (2 × 10^6^/plate CFU). Only very few CFU were observed in the case of *S. aureus* and particularly of *K. pneumoniae* when these strains were initially seeded to the highest concentrations. No photoreactivation was observed in this study (Table 1, Figure S2).

The kinetics of the UV-C irradiation data (Table 2) demonstrate that a bacterial number reduction of 5 logarithmic units can be obtained by an irradiation dose of 9.66 mJ/cm^2^ for all tested strains. The photoreactivation response of bacteria was assessed on culture plates exposed to light for 4 h after UV-C irradiation. Time-course data showed no significant differences in terms of the time of UV-C irradiation required to reach the antimicrobial activity observed for the different strains (Table 2). For *K. pneumonia*, even if a high number of CFU was observed after photoreactivation, the difference was not significant. However, after 22.97 mJ/cm^2^ of irradiation, no photoreactivation was detected.

### 3.3. Bacterial Inactivation of Static UV-C Irradiation on Different Materials

The bactericidal activity of UV-C (9.66 mJ/cm^2^) was studied on different materials (i.e., glass, plastic, steel) that compose HTSs where bacterial suspensions (2 × 10^6^/mL) were spread (Table 3). The results confirmed the reduction of the CFU number by 5 log units observed earlier (Table 1) in all three materials; however, some specificities were observed in the different strains. In detail, the exposure of *S. aureus* ATCC^®^ 25923^TM^ layered on glass material to UV-C light resulted in complete inactivation (average residual count of 1 ± 1 CFU), while plastic and steel showed 16.0 ± 7.8 and 15.3 ± 6.7 residual CFU, respectively. The MRSA strain was inactivated more efficiently on steel (3.0 ± 1.7 CFU) than on glass or plastic (22.3 ± 12.7 and 24.3 ± 4.7 CFU, respectively). On the *P. aeruginosa* PAO-1 strain, UV-C light was more effective on glass-seeded bacteria than on plastic and steel (1.7 ± 0.6 vs. 46.7 ± 48.0 and 46, 0 ± 10.1, respectively). On the other hand, for the *P. aeruginosa* strain ATCC^®^ 27853^TM^, the irradiation showed the same effect on the three materials under examination (2.7 ± 1.5 on plastic, 0.7 ± 1.1 on glass, and 0.7 ± 0.6 on steel). Finally, for the two strains of *K. pneumoniae* KPC 242 and 260 resistant to carbapenems, UV-C irradiation was more active in reducing the CFU number on glass (5.7 ± 5.0 for KPC 242 and 0.0 ± 0.0 for KPC 260) than on plastic (19.7 ± 11.7 for KPC 242 and 42.3 ± 34.5 for KPC 260) and steel (58.3 ± 43.9 for KPC 242 and 30.7 ± 18.6 for KPC 260).

## 4. Discussion

In this study, we demonstrated that static UV-C irradiation produces a bactericidal effect against multi-antibiotic resistant clinical isolates, including *S. aureus* MRSA, *P. aeruginosa*, and *K. pneumoniae* KPC, with a reduction of CFU number up to 6 logarithmic units. Irradiation of 12.68 mJ/cm^2^ was effective in all these clinical isolates, including multi-resistant strains, which were abated by 5 logarithmic units. Comparable results have already been reported for *S. aureus* ATCC*^®^* 25923^TM^ [10], *S. aureus* MRSA [11], *P. aeruginosa*, and *K. pneumoniae* [13,14,15,16]. Furthermore, we found that the proposed static irradiation protocol also prevents bacterial photoreactivation [17], thus excluding a bacteriostatic effect by the UV-C light. Again, we first demonstrated that the static irradiation of 9.66 mJ/cm^2^ inactivates these different stains by up to 5 log units on three different types of materials of HTSs, including glass, plastic, and steel. Plastic appears to represent the most difficult material to sanitize in comparison with the others investigated in this study, especially glass, which, apart from for *S. aureus* MRSA, was found to present the lowest number of CFU after UV-C irradiation. These differences could be explained by the fact that plastic is characterized by more porous and irregular surfaces than glass and steel, also favoring the persistence upon UV-C irradiation of other microbiological agents including SARS-CoV-2 [18]. Important enough is that in these experiments we utilized very high concentrations of multi-drug resistant bacteria. These are expected to exceed the actual bacterial concentrations of the surfaces of the hospital environment by different orders of magnitude (after ordinary cleaning, the number of colonies observed upon microbiological examination usually does not exceed 200 CFU per plate). Therefore, the observed data demonstrate a bactericidal activity of the UV-C light at short exposure times even when a very high bacterial load is present. Therefore, these findings suggest applications for UV-C irradiation in procedures where these strains can be present as a potential cause of infections on different surfaces for which accurate and periodic sanitation is needed. It is worth noting that dynamic disinfection tests were proven to be much more efficient and reproducible than manual disinfection procedures in use at the hospital facilities investigated in this study, which consisted of the manual cleaning of furniture and floors followed by nebulization with chlorine. The efficacy of the robotic device utilized in this study could also be explained by the innovation introduced in the mobile irradiation technique. The device is equipped with an array of 254 nm UV-C lamps that provide the widest efficacy of surface irradiation among the mobile devices of this type produced and commercialized so far. According to these specificities, during the tests performed with the R2S device, efficient bacterial inactivation was obtained in the case of “blinded”, or partially hidden, surfaces, such as the lower surface of the right armrest of a patient’s chair. The possibility that the device may operate on reflecting materials could further improve its bactericidal potency that is already very high. The UV-C dose was measured by using a UV-C dosimeter, a 15 mm diameter dot of self-adhesive substrate containing a reactive indicating ink calibrated to experience different color changes depending on the accumulated radiation dose received. During the R2S mission the dosimeters, placed near the sampled surfaces, detected an irradiation intensity between 75 and 100 mJ/cm^2^; doses much higher than those used in the experiments with the fixed lamp were observed. These results suggest that a shorter irradiation time could be used. Again, this type of device is equipped with artificial intelligence and monitoring technologies that may optimize and verify the disinfection mission and its correspondence with the sanitization strategy selected for each environment, including intentional or accidental interruptions of operativity and respect of safety procedures. It has been demonstrated that exposure to UV-C light can provoke eye and skin damage. The R2S robot works in closed rooms and is equipped with a sensor-based safety shutdown of UV-C lamps on the neural network “Human Body Recognition”. The sensor ensures the system will not operate if a person enters the space during a disinfection cycle. This feature makes the R2S system safe to use in hospital environments. However, UV-C treatment does not replace basic cleaning; rather, this method can represent a complementary strategy to manual cleaning, which is necessary to eliminate dust and organic residues. Furthermore, the results indicate that the repeated use of this robotic device in the same room may help to reduce the number of microbes present before sanitizing HTSs that are more difficult to sanitize with other procedures, such as computer keyboards and mice.

## 5. Conclusions

The observed data suggest that disinfection protocols of UV-C, especially dynamic (or mobile) ones, provide an efficient means to sanitize different surfaces, thus representing a valid alternative to manual cleaning and their limits of efficacy in nosocomial sanitization protocols. Regarding the current issue of innovation in disinfection protocols, the use of robots could reduce human errors, frequent in routine work, and decrease pollution due to biocides and the disposal of the different consumables used in manual cleaning.

## Figures and Tables

**Table 1:** Bacterial inactivation and photoreactivation after in vitro UV-C irradiation.

	Baseline	CFU at Seeding	Inactivation Test * CFU/Plate	Photoreactivation Test CFU/Plate
***S. aureus*** ***ATCC*** ***^®^*** ***25923^TM^***	2 × 10^4^	1.4 × 10^4^ ± 3.8×10^3^	0.0 ± 0.0	0.0 ± 0.0
	2 × 10^5^	1.6 × 10^5^ ± 5.9 × 10^4^	0.3 ± 0.6	0.3 ± 0.6
	2 × 10^6^	1.9 × 10^6^ ± 5.7 × 10^5^	1.3 ± 1.2	2.7 ± 4.6
***S. aureus MRSA***	2 × 10^4^	2.2 × 10^4^ ± 2.8 × 10^2^	0.0 ± 0.0	0.0 ± 0.0
	2 × 10^5^	4.9 × 10^5^ ± 9.3 × 10^4^	2.0 ± 3.5	0.7 ± 0.6
	2 × 10^6^	1.9 × 10^6^ ± 8.4 × 10^5^	1.3 ± 0.6	0.7 ± 1.2
***P. aeruginosa*** ***ATCC*** ***^®^*** ***27853^TM^***	2 × 10^4^	ND	ND	ND
	2 × 10^5^	3.4 × 10^5^ ± 9.8 × 10^4^	0.0 ± 0.0	0.0 ± 0.0
	2 × 10^6^	1.9 × 10^6^ ± 4.8 × 10^5^	0.0 ± 0.0	0.0 ± 0.0
***P. aeruginosa*** ***PAO-1***	2 × 10^4^	5.2 × 10^4^ ± 2.8 × 10^3^	0.0 ± 0.0	0.0 ± 0.0
	2 × 10^5^	2.8 × 10^5^ ± 2.3 × 10^4^	0.0 ± 0.0	0.7 ± 1.2
	2 × 10^6^	2.7 × 10^6^ ± 7.8 × 10^4^	0.0 ± 0.0	2.0 ± 2.0
***K. pneumoniae*** ***KPC242***	2 × 10^4^	8.5 × 10^3^ ± 2.2 × 10^3^	0.0 ± 0.0	0.0 ± 0.0
	2 × 10^5^	1.5 × 10^5^ ± 5.2 × 10^4^	8.7 ± 9.0	1.7 ± 2.1
	2 × 10^6^	1.8 × 10^6^ ± 4.9 × 10^5^	7.0 ± 11.2	16.7 ± 8.1
***K. pneumoniae*** ***KPC260***	2 × 10^4^	6.4 × 10^3^ ± 2.7 × 10^2^	0.0 ± 0.0	0.0 ± 0.0
	2 × 10^5^	2.3×10^5^ ± 6.0 × 10^4^	1.3 ± 1.2	1.3 ± 1.2
	2 × 10^6^	2.2 × 10^6^ ± 4.2 × 10^5^	6.7 ± 11.5	14.7 ± 8.3

* Inactivation was assessed after a time of exposure to 12.68 mJ/cm^2^ of UV-C for *S. aureus* and *P. aeruginosa*, and 22.97 mJ/cm^2^ of UV-C for *K. pneumonia* strains. ND: Not determined.

**Table 2:** Kinetics of bacterial inactivation and photoreactivation after in vitro UV-C irradiation.

	Irradiation (mJ/cm^2^)	UV-C Exposure CFU/Plate	Photoreactivation CFU/Plate
***S. aureus*** ***ATCC*** ***^®^*** ***25923^TM^***	0	1.6 × 10^5^ ± 5.9 × 10^4^	
	4.33	269.3 ± 249.8	330.0 ± 304.1
	9.66	8.7 ± 15.0	0.0 ± 0.0
	12.68	0.3 ± 0.6	0.0 ± 0.0
***S. aureus MRSA***	0	4.9 × 10^5^ ± 9.2 × 10^4^	
	4.33	2.0 ± 3.5	3.0 ± 5.2
	9.66	3.3 ± 5.8	0.0 ± 0.0
	12.68	0.3 ± 0.6	0.0 ± 0.0
***P. aeruginosa*** ***ATCC*** ***^®^*** ***27853^TM^***	0	3.3 × 10^5^ ± 9.8 × 10^3^	
	4.33	NT	NT
	9.66	NT	NT
	12.68	0.0 ± 0.0	0.0 ± 0.0
***P. aeruginosa PAO-1***	0	2.8 × 10^5^ ± 2.2 × 10^4^	
	4.33	0.0 ± 0.0	41.00 ± 69.3
	9.66	0.0 ± 0.0	0.0 ± 0.0
	12.68	0.0 ± 0.0	0.0 ± 0.0
***K. pneumoniae KPC242***	0	1.5 × 10^5^ ± 5.2 × 10^4^	
	4.33	535.0 ± 379.6	592.7 ± 375.0
	9.66	1.3 ± 1.2	13.3 ± 15.3
	22.97	8.7 ± 9.0	1.7 ± 2.1
***K. pneumoniae KPC260***	0	2.3 × 10^5^ ± 6.0 × 10^4^	
	4.33	508.0 ± 426.14	567.67 ± 417.9
	9.66	1.3 ± 1.2	38.0 ± 32.7
	22.97	1.3 ± 1.2	1.3 ± 1.2

**Table 3:** Effect of UV-C irradiation on different materials.

	Exposure mJ/cm^2^	CFU/ at Seeding	Sample Surface Irradiation	CFU/Plate After Exposure		
				Plastic	Glass	Steel
***S. aureus* ATCC25923**	9.66	2.1 × 10^6^ ± 2.7 × 10^5^	Aluminium covered	2.1 × 10^6^ ± 9.9 × 10^4^	2.0 × 10^6^ ± 2.0 × 10^5^	2.0 × 10^6^ ± 2.4 × 10^5^
			Uncovered	16.0 ± 7.81 **	1.00 ± 1.00 **	15.33 ± 6.66 **
***S. aureus MRSA881***	9.66	2.2 × 10^6^ ± 3.9 × 10^5^	Aluminium covered	2.1 × 10^6^ ± 2.8 × 10^5^	2.2 × 10^6^ ± 4.8 × 10^4^	1.9 × 10^6^ ± 2.3 × 10^5^
			Uncovered	24.33 ± 4.73 *	22.33 ± 12.70 *	3.00 ± 1.73 *
***P. aeruginosa* ATCC7853**	9.66	1.7 × 10^6^ ± 3.1 × 10^5^	Aluminium covered	1.7 × 10^6^ ± 1.1 × 10^5^	1.6 × 10^6^ ± 6.4 × 10^4^	1.7 × 10^6^ ± 1.1 × 10^4^
			Uncovered	2.67 ± 1.53 *	0.67 ± 1.15 *	0.67 ± 0.58 *
***P. aeruginosa PAO1***	9.66	1.9 × 10^6^ ± 3.7 × 10^5^	Aluminium covered	1.8 × 10^6^ ± 8.5 × 10^4^	1.9 × 10^6^ ± 7.0 × 10^4^	1.9 × 10^6^ ± 1.5 × 10^5^
			Uncovered	46.67 ± 48 *	1.67 ± 0.58 *	46.00 ± 10.15 *
***K. pneumoniae KPC 242***	9.66	2.0 × 10^6^ ± 4.1 × 10^5^	Aluminium covered	2.0 × 10^6^ ± 2.1 × 10^5^	2.0 × 10^6^ ± 1.6 × 10^5^	2.0 × 10^6^ ± 1.4 × 10^5^
			Uncovered	19.67 ± 11.68 *	5.67 ± 5.03 *	58.33 ± 43.88 *
***K. pneumoniae KPC 260***	9.66	2.4 × 10^6^ ± 4.5 × 10^5^	Aluminium covered	2.3 × 10^6^ ± 2.8 × 10^5^	2.2 × 10^6^ ± 3.6 × 10^5^	2.2 × 10^6^ ± 4.0 × 10^5^
			Uncovered	42.33 ± 34.49*	0.00 ± 0.00 *	30.67 ± 18.58 *

Aluminium covered surfaces were sampled by mean of swabs diluted in MHB and spread on MHA plates. * *p* < 0.05, ** *p* < 0.001, CFU after exposure vs CFU at seeding.

## Data Availability

For details regarding our data supporting reported results can be contact the corresponding authors.

## Supplementary Materials

The following are available online at https://www.mdpi.com/article/10.3390/ijerph182413019/s1: Supplementary Figure S1: Comparison of RODAC plates and Swab sampling methods; Supplementary Figure S2: UVC irradiation kinetics and its effect on bacterial photoreactivation; https://zenodo.org/record/5512515#.YbKuNtDP2Um: Supplementary Video S1: R2S mission.
