# Supplementary material for: Effect of a UV-C Automatic Last-Generation Mobile Robotic System on Multi-Drug Resistant Pathogens
Source: Int J Environ Res Public Health. 2021 Dec 10;18(24):13019. doi: 10.3390/ijerph182413019 (PMC8701089; doi:10.3390/ijerph182413019)
Supplement: Supplementary file 1 [file ijerph-18-13019-s001.zip › ijerph-1487825-supplementary.pdf]

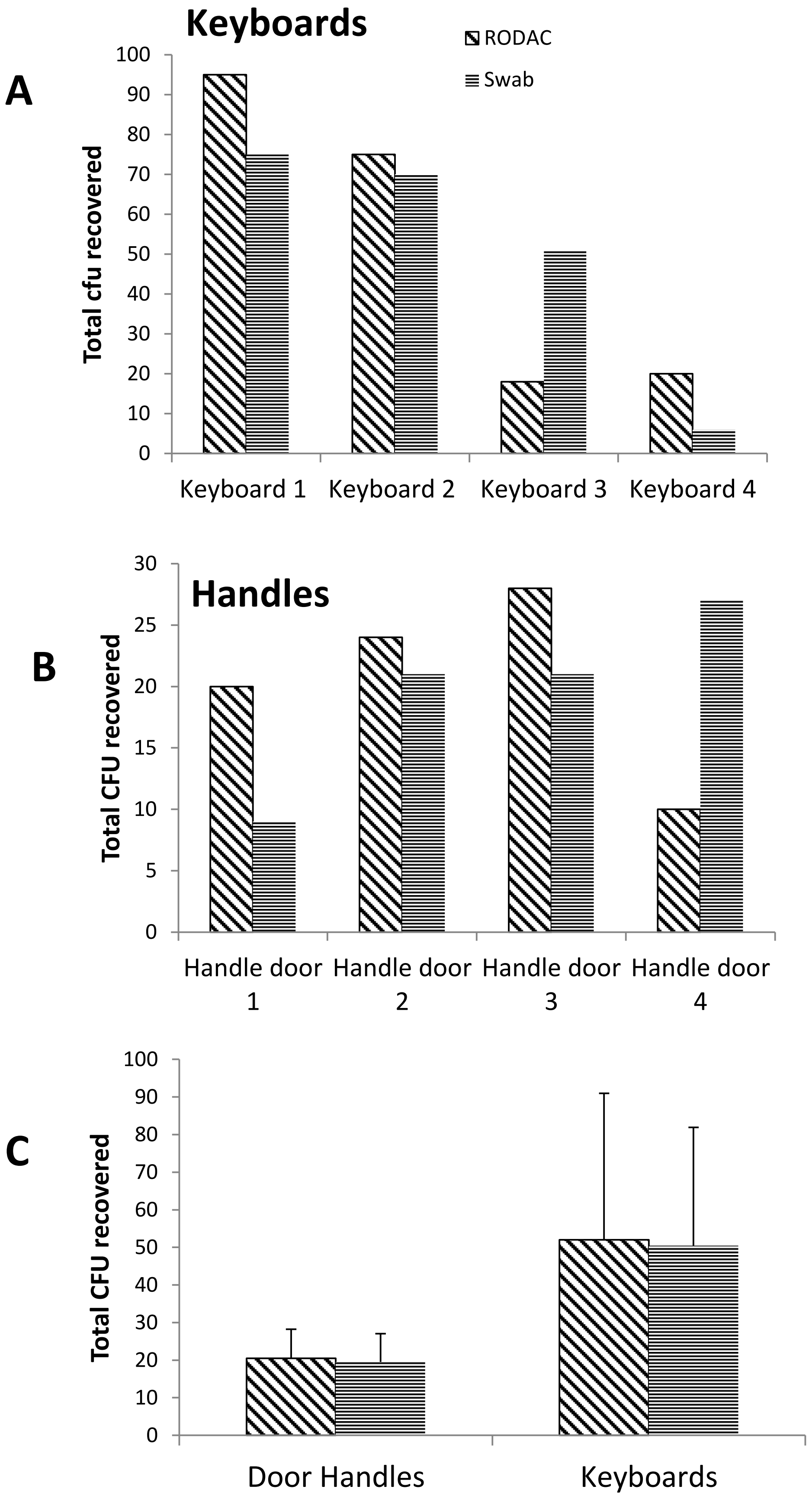

Supplementary Figure S1. Comparison of RODAC plates and Swab sampling methods. Microbial load of 4 different keyboards (**A**) and 4 different handles (**B**) were determined with RODAC plates method and swab sampling. The recovered CFU mean±SD were reported in (**C**).

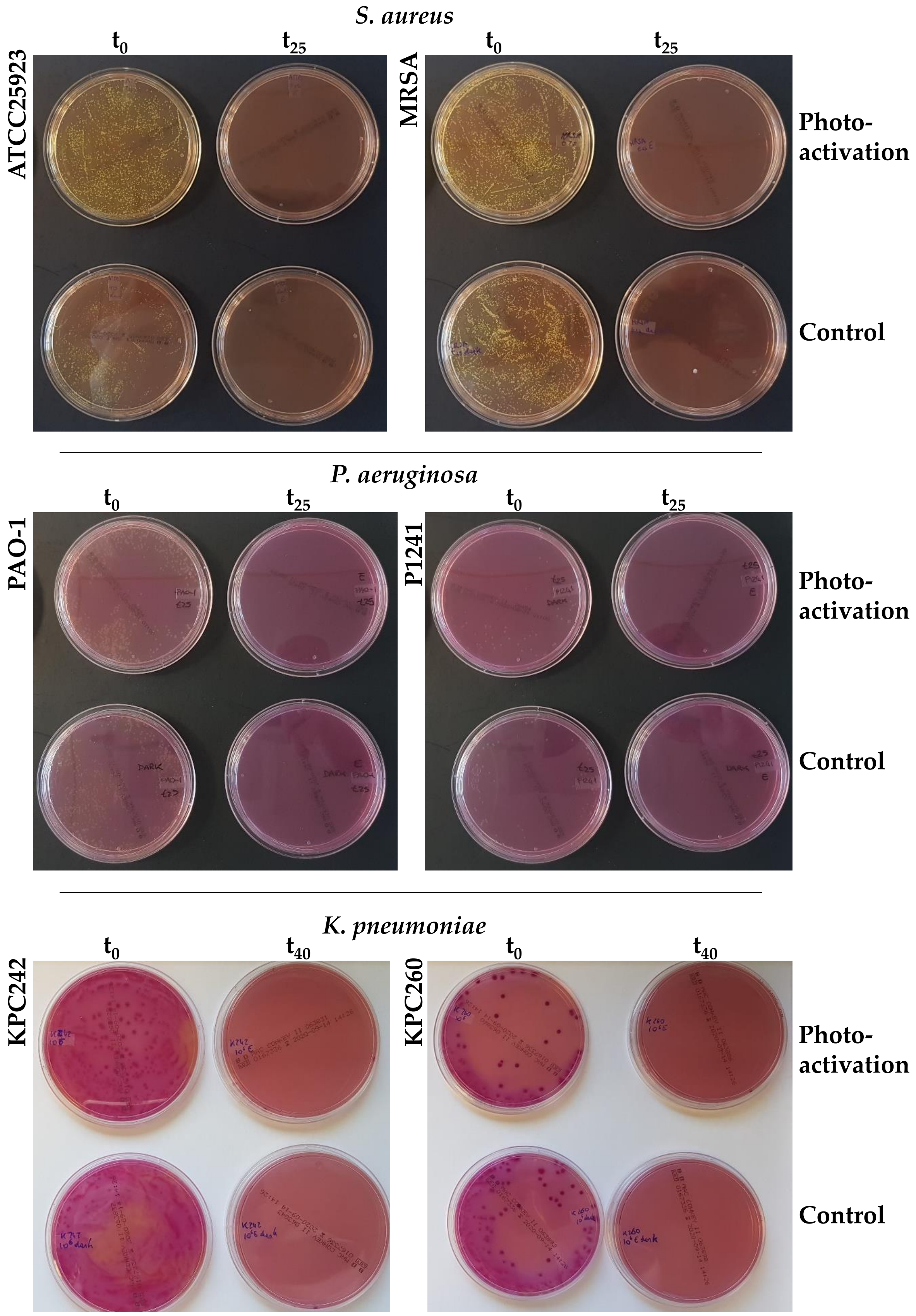

Supplementary Figure S2: UVC irradiation kinetics and its effect on bacterial photoreactivation. The tests were performed starting from a bacterial density of $2x10^5$ CFU/plate, and irradiation time was set at 25 seconds for *S. aureus* ATCC25923 and *S. aureus* MRSA and for *P. aeruginosa*, and at 40 seconds for *K. pneumoniae*; other details are described in the legend to Figure 1 and the section “Methods”.
